# Epigenetic loss of heterozygosity of *Apc* and an inflammation-associated mutational signature detected in *Lrig1*^*+/−*^*-*driven murine colonic adenomas

**DOI:** 10.1186/s12885-020-6616-y

**Published:** 2020-02-14

**Authors:** Jessica L. Preston, Nicholas Stiffler

**Affiliations:** Institute of Molecular Biology, University of Oregon, Eugene, OR 97403 USA

**Keywords:** *Lrig1*, *Lgr5*, Colorectal cancer, Intestinal stem cells, Adenoma, Mutations

## Abstract

**Background:**

The loss of a single copy of adenomatous polyposis coli (*Apc)* in leucine-rich repeats and immunoglobulin-like domains 1 (*Lrig1)-*expressing colonic progenitor cells induces rapid growth of adenomas in mice with high penetrance and multiplicity. The tumors lack functional APC, and a genetic loss of heterozygosity of *Apc* was previously observed.

**Methods:**

To identify genomic features of early tumorigenesis, and to profile intertumoral genetic heterogeneity, tumor exome DNA (*n* = 9 tumors) and mRNA (*n* = 5 tumors) sequences were compared with matched nontumoral colon tissue. Putative somatic mutations were called after stringent variant filtering. Somatic signatures of mutational processes were determined and splicing patterns were observed.

**Results:**

The adenomas were found to be genetically heterogeneous and unexpectedly hypermutated, displaying a strong bias toward G:C > A:T mutations. A genetic loss of heterozygosity of *Apc* was not observed, however, an epigenetic loss of heterozygosity was apparent in the tumor transcriptomes. Complex splicing patterns characterized by a loss of intron retention were observed uniformly across tumors.

**Conclusion:**

This study demonstrates that early tumors originating from intestinal stem cells with reduced *Lrig1* and *Apc* expression are highly mutated and genetically heterogeneous, with an inflammation-associated mutational signature and complex splicing patterns that are uniform across tumors.

## Background

Human colorectal cancer (CRC) is the second leading cause of cancer death in the US, and ~ 25% of patients with CRC are incurable at the time of diagnosis [1, 2]. Genetic heterogeneity is inherent to this disease, providing tumor cells with the ability to rapidly adapt and resist treatments [3, 4]. Currently CRC is clinically segregated into four broad subcategories based on the expression of various biomarker molecules called consensus molecular subtypes (CMS1–4) [5]. Generally, CMS1 tumors display microsatellite instability and immune activation, CMS2 tumors are epithelial and display WNT pathway activation, CMS3 tumors are KRAS-driven, and CMS4 tumors are mesenchymal with VEGF activation. However, this classification system oversimplifies the diversity and interrelatedness of cancer cell subtypes. The specific steps required to counteract the critical aspects of CRC progression remain poorly understood despite decades of research.

The cell-of-origin of CRC derives from a population of rapidly-dividing stem cells located at the base of the colonic epithelial crypts, which are identifiable based on the expression of Leucine-rich repeat containing G protein-coupled receptor 5 (*Lgr5*). *Lgr5* is the downstream target of *R-spondin* in the canonical Wnt/β-catenin pathway. Mutations in the tumor-suppressor gene adenomatous polyposis coli (*Apc*) and other members of the canonical Wnt pathway are the hallmark of CRC [6–9]. Loss of heterozygosity (LOH) of *Apc* tends to occur during the early stages of human CRC tumorigenesis.

The standard mouse model used for CRC research, *Apc*^Min/+^, contains a truncating point mutation in one copy of *Apc.* It is believed that most *Apc*^Min/+^ tumors have lost *Apc* function through a spontaneous genetic LOH [10]. *Apc*^Min/+^ mice exhibit tumor formation predominately in the small intestine rather than in the distal colon as observed in humans. Interestingly, Tanaka, T., et al. 2006 reported that dextran sodium sulfate (DSS)-induced inflammation increased the incidence of polyps in the distal colon of *Apc*^Min/+^ mice [11]. Similarly, Yang, K., et al. 2008 found that reduced mucus production in *Apc*^Min/+^ mice shifted tumor development toward the distal colon. In 2009 Ritchie, K., et al. crossed a glutathione S-transferase Pi (*Gstp)* null allele into the standard *Apc*^Min/+^ mouse and reported a 6-fold increase in distal colorectal adenoma incidence and a 50-fold increase in adenoma multiplicity compared to *Apc*^*Min*^ mice [12]. The authors also noted that the colons of the (*Gstp)* null *Apc*^Min/+^ mice expressed higher levels of inflammatory molecules interleukin 4 *(IL4)*, interleukin 6 *(IL6),* and nitric oxide synthase. Taken together, these results indicate an important role for mucus in reducing inflammation-associated tumors in the distal colon [13].

Leucine-rich repeats and immunoglobulin-like domains 1 (*Lrig1)* is a transmembrane feedback regulator of growth factor receptor tyrosine kinases that is expressed in the *Lgr5*^+^ stem cell population present at the base of colonic crypts [14–17]. *Lrig1* acts as a tumor-suppressor gene in several contexts [18–23]. The *Lrig1-CreERT2/+;Apcfl/+* inducible mouse model of colonic adenoma is based on the conditional Cre-recombinase-driven loss of a single copy of *Apc* under the control of the *Lrig1* promoter [24, 25]*.* The colonic stem cells of these mice express one single wild type copy each of the *Lrig1* and *Apc* genes after the engineered recombination of *Apc* in *Lrig1*^*+/−*^-expressing stem cells. Within 100 days of the loss of one copy of *Apc*, rapidly growing adenomas appear in the distal colon with extremely high tumor penetrance and multiplicity. The *Lrig1-CreERT2/+;Apcfl/+* CRC model is very similar to the *Gstp*-null*;Apc*^*Min*^ CRC model in terms of tumor onset, penetrance, multiplicity, anatomical location, and mortality. These findings imply that a common mechanism involving inflammation-induced tumor formation is responsible for tumorigenesis in the distal colon.

Human tumors often exhibit distinct patterns of mutations that can provide clues into the origin and mechanism of tumorigenesis. The ‘somatic signature of mutations’ of a tumor is based on the specific nucleotide alterations present and the background sequence context of the mutations [26–28]. Somatic signatures are influenced by specific carcinogenic agents and DNA repair genes, and can therefore sometimes reveal sources of mutation and mechanisms of tumorigenesis. For example, C > A transversion point mutations typically occur in low frequencies compared to C > T transition point mutations. However, lung and esophageal tumors caused by tobacco tar often contain an abundance of C > A transversions due to the conjugation of nitrosamines to glutathione, which forms guanine adducts [29]. Stomach cancers caused by *H. pylori* infection also contain a high incidence of C > A transversions, presumably due to the inflammation-associated reactive oxygen and nitrogen species (ROS and RNS) [30].

This work sought to understand the genomic changes occurring during the early stages of tumorigenesis in rapidly-growing colonic adenomas in *Lrig1-CreERT2/+;Apcfl/+* mice. Exomic DNA and mRNA sequences from the adenomas were analyzed in order to detect the presence of transcriptomic and genomic alterations. Specifically, the genetic heterogeneity across tumors and somatic signatures of mouse colonic adenomas tumors were assessed using exome DNA profiling, and the prevalence of differential gene expression and splicing defects was assessed using mRNA-Seq.

## Materials and methods

### Generation of Lrig1-driven colonic adenomas

Generation of *Lrig1-CreERT2/+;Apcfl/+* tumors was previously described [25]. Briefly, *Lrig1-CreERT2/+* mice were crossed to Apc580S*/+* mice (Jackson Laboratory, Bar Harbor, ME, U.S.A) [31] to generate *Lrig1-CreERT2/+;Apcfl/+* mice [24]. Adult (6- to 8-week-old) *Lrig1-CreERT2/+;Apcfl/+* mice were intraperitoneally injected with 2 mg tamoxifen per mouse (Sigma-Aldrich, St. Louis, MO, U.S.A.) in corn oil for 3 consecutive days and multiple dysplastic colonic adenomas were extracted 100 days later. For the DNA studies, the control is nontumor tissue parts of the same *Lrig1-CreERT2/+;Apcfl/+* mouse, referred to as ‘nontumor’ in figures. For the RNA studies, the control is untreated wild type C57BL/6 J mice, referred to as ‘wild type’ in figures.

### Exomic DNA sequencing

Exomic DNA sequencing (*n* = 9 tumors) was performed by HudsonAlpha Labs (Huntsville, AL, U.S.A.) Exome capture using NimbleGen v3.0 captured 64 megabasepair (Mbp) baits. A total of six gigabasepairs (Gbp) of exome data were sequenced per sample. Exomic tumor DNA of nine tumors from three mice was sequenced in parallel with adjacent normal colon (*n* = 3) to 12x average read depth. Adjacent normal colon tissue was used for the ‘nonutmor’ control.

### DNA read alignment and processing

Raw fastq reads were cleaned to remove low quality bases at the ends of the reads using Stacks (v 1.35) process_shortreads [32]. Cleaned reads were aligned to the *Mus musculus* (house mouse) genome (2011 assembly, UCSC Genome Browser assembly ID mm10, Genome Reference Consortium Mouse Build 38, Accession GCA_000001635.2) using Bowtie2 (v2.2.1), in default-sensitive mode [33]. Sam/bam files were sorted and indexed using SAMtools (v0.1.18) [34] and Picard Tools (v1.92) (Broad Institute, Cambridge, MA, U.S.A.). Base quality score recalibration and indel realignment were performed according to GATK best practices [35].

### Mutation calling, filtering, and visualization

Somatic mutations were called using SeuratSomatic (v2.5), a GATK module [36–38]. Somatic variants were called using a minimum variant coverage of 4 reads and minimum quality score of Q10. To ensure that the identified mutations were somatic in nature, matched normal tissue samples were used to filter germline variants. Residual germline polymorphisms were removed by filtering all germline variants reported on the National Center for Biotechnology Information (U.S.A.) dbSNP database [39]. To reduce the occurrence of sequencing errors in the data, variants were called only when present in both the forward and reverse DNA strand [40]. To confirm that the discovered mutations were not due to alignment artifacts, the presence of representative variants was verified in the raw sequencing reads using grep and BLAST. The predicted effect of the mutations on protein function was determined with Ensembl’s variant effect predictor (VEP) program [41] as well as the SNPeff variant annotation and effect prediction tool [42]. The somatic signature of the mutations was identified using R package SomaticSignatures [38]. Genomic data was visualized using R packages GenVisR and ggplot2 [43, 44] Negative control nontumor samples and mouse glioblastoma whole-genome DNA sequencing datasets (*n* = 3, provided by Hui Zong Lab, University of Virginia Medical School) were compared to the adenoma dataset; hypermutation or a clear somatic signature were not observed in the negative controls.

### RNA sequencing and analysis

Whole tumors (*n* = 5) as well as normal wild type colonic tissue from untreated C57BL/6 J mice (*n* = 3), were assayed. RNA was sequenced to 25x average read depth. Cleaned cDNA reads were aligned to the mouse mm10 genome (Accession GCA_000001635.2) with STAR (v1.0) [45]. Changes in gene expression were identified using R package DESeq2 and visualized with R packages DESeq2, ggplot2, and Pathview. A false discovery rate (FDR) < 0.1 was used as the significance threshold. Custom bigwig tracks were created on the University of California at Santa Cruz Genome Browser in order to assess splicing patterns and copy number variations [46].

## Results

### Tumor exome mutations

**Fig. 1:**
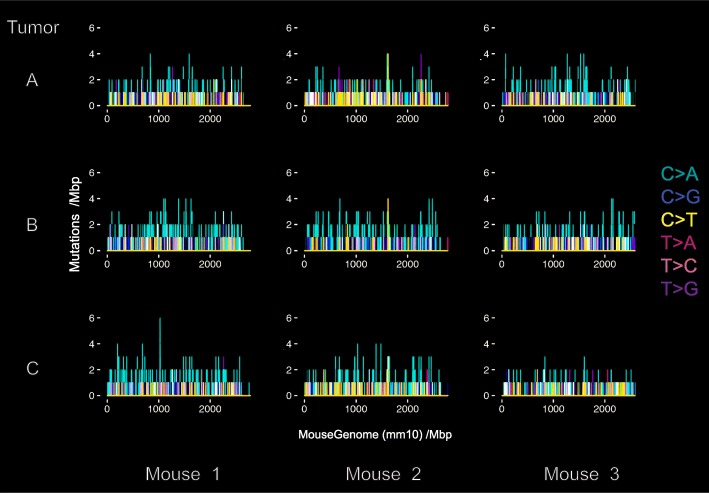
Colonic adenomas resulting from inducible loss of adenomatous polyposis coli (*Apc*) in leucine-rich repeats and immunoglobulin-like domains 1 (*Lrig1*)+/− stem cells are hypermutated and genetically heterogeneous. Exome sequencing of adenomas from *Lrig1*-(*Apc*-Flox) mice detected the presence of ~25 to 35 high-quality somatic mutations per megabase, with an abundance of C:G>A:T transversion point mutations. The number and type of somatic mutations from each adenoma exome is plotted. There were nine adenomas from three mice; each column plots three tumors from a single mouse. Similar mutational frequencies and motif distributions were observed across the nine tumor exomes sequenced

Adenomas resulting from the inducible loss of one copy of *Apc* in *Lrig1*^+/−^-expressing colonic stem cells are hypermutated and genetically heterogeneous. Tumor exome DNA contained 930–1300 high-quality somatic mutations per tumor (~ 25–30 mutations per megabase) distributed uniformly throughout the genome. Variants were called only when present in both the forward and reverse DNA strand, with a minimum quality score of Q10 and a minimum variant coverage of 4 reads. The tumors are highly heterogeneous in terms of the specific genetic loci mutated, however nearly identical frequencies of mutated nucleotide motifs were observed across all nine tumors sequenced (Fig. 1). No large copy number variations or rearrangements were detected.

**Fig. 2:**
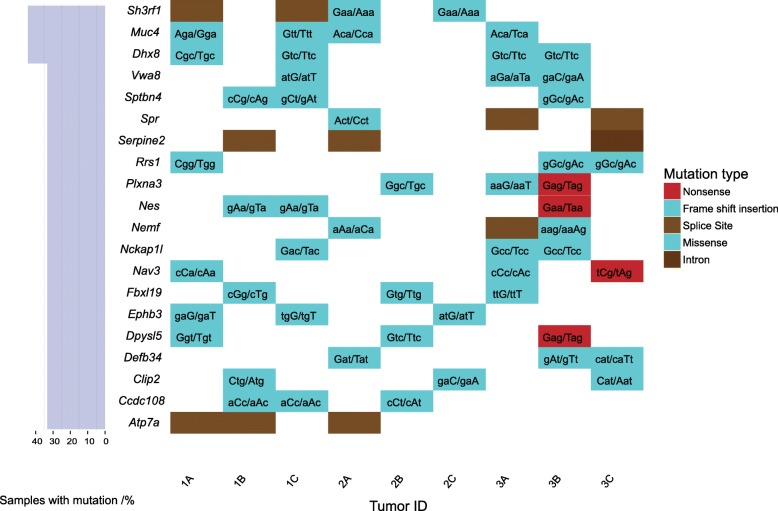
Putative driver mutations in colonic adenomas resulting from inducible loss of adenomatous polyposis coli (*Apc*) in leucine-rich repeats and immunoglobulin-like domains 1 (*Lrig1*)+/− stem cells. Two genes were mutated in 4/9 tumors and 11 genes were independently mutated in >30% of the tumors; these are shown as rows. Each column represents a single colonic tumor. The majority of the mutations were missense, C:G>A:T transversions. This plot was created using GenVisR [44]

Each adenoma contained a unique profile of ~ 100 high-frequency and high-quality mutations predicted to cause significant functional disruptions based on amino acid conservation (Table S1**)**. Two genes were mutated in 4/9 tumors: mucin 4 *(Muc4)* and DEAH-Box Helicase 8 (*Dhx8). Dhx8* is involved in alternative splicing. Eleven genes were independently mutated in >30% of the tumors (Fig. 2, Table S2). The 40 most commonly mutated genes (>20%) are predicted to affect cell morphology and migration based on pathway analysis, and were found to be mutated in 10–20% of human CRC cases in The Cancer Genome Atlas (TCGA) database.

Mutated tumor-suppressor genes, such as phosphatidylinositol-4,5-bisphosphate 3-kinase catalytic subunit alpha (*Pi3kca),* Neurofibromin 1 *(Nf1),* and *Lrig1* were observed. The DNA repair genes *MutS* protein homolog 4 (*Msh4),* MutS protein homolog 6 *(Msh6),* and DNA polymerase delta 1 *(Pold1)* were also mutated. Unexpectedly, most tumors contained several additional mutations in genes of canonical and non-canonical *Wnt* pathways. The specific genes affected were different for each of the tumors.

### Tumor somatic signatures

**Fig. 3:**
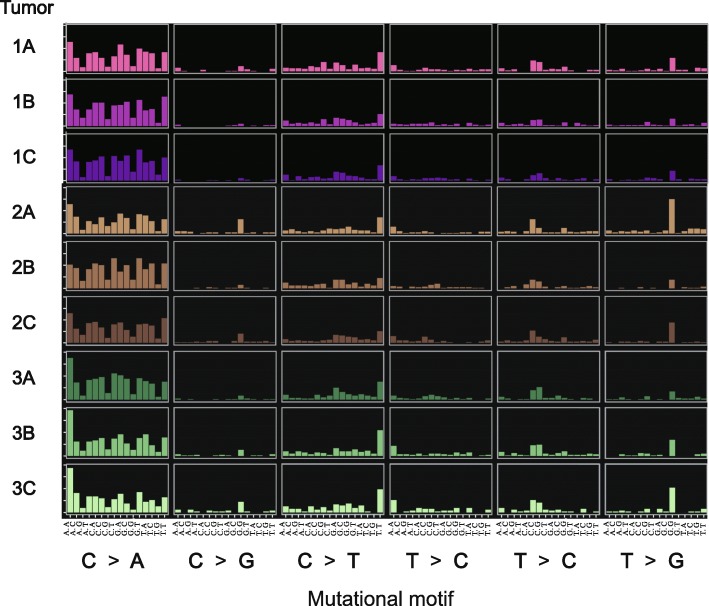
Colonic adenomas resulting from inducible loss of adenomatous polyposis coli (*Apc*) in leucine-rich repeats and immunoglobulin-like domains 1 (*Lrig1*)+/− stem cells have a distinct pattern of somatic mutations dominated by C:G>A:T transversion point mutations. The somatic signature of mutations of the adenomas are plotted as rows; there were nine adenomas from three mice. Each adenoma was found to contain a nearly identical pattern of somatic mutations based on motifs and background sequence context. The somatic signature is characterized by an abundance of C to A transversion point mutations, which are caused by defects in repairing DNA adducts of guanine and are frequently observed in cancer associated with tobacco tar and Helicobacter pylori infections

The adenomas exhibited an overwhelming abundance of C to A transversion point mutations at remarkably similar frequencies. The somatic mutations displayed nearly identical patterns of background genomic sequence context across the nine tumors (Fig. 3). This type of mutational signature is often seen in smoking-induced lung cancer and stomach cancer induced by *Helicobacter pylori* infection, which are associated with an increase in the formation of guanine adducts [27, 28]. The high incidence of C to A transversions observed in the tumor exomes implies that the loss of one copy of *Apc* in *Lrig1*^+/−^ colonic stem cells increased inflammation and led to the formation of DNA adducts due to the oxidation of guanine.

### Tumor transcriptomes

**Fig. 4:**
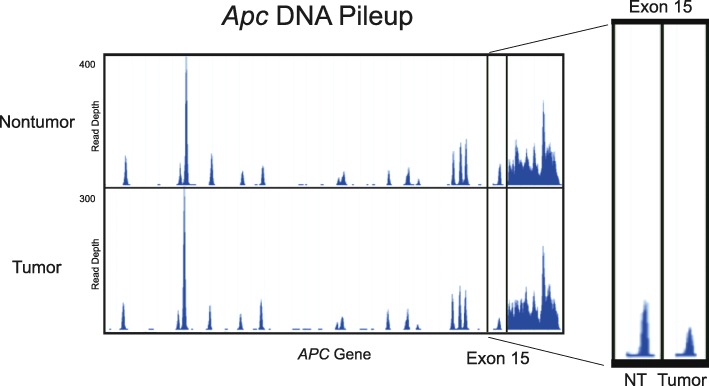
The loss of heterozygosity of the adenomatous polyposis coli (*Apc*) gene was not observed through DNA sequencing. Colonic adenomas resulting from inducible loss of *Apc* in leucine-rich repeats and immunoglobulin-like domains 1 (*Lrig1*)+/− stem cells are missing only one copy of exon 15 of the *Apc* gene. RNA sequencing reads from adjacent nontumor tissue (top row) and adenoma (bottom row) are plotted as pooled pileups. Allelic loss is typically considered to occur when the amount of tumor DNA was 50% or less than the value obtained from heterozygous tissue, due to the high risk of contamination from wild-type cells. Here, the sequenced tumor cells containend ~50% of the adjacent normal DNA which was assumed to be homozygous for wild-type *Apc*. No additional somatic mutations in *Apc* were observed across the nine tumors including genetic deletions, although many other Wnt/β- catenin genes were mutated

Despite their highly variable genomes, the tumors exhibited substantial transcriptome similarity to each other (Fig. 4). Down-regulation of the DNA repair genes *Msh3* (FDR = 0.57) and *Msh4* (FDR = 0.026) were observed (Table S3). In addition, eight UDP glucuronosyltransferases were downregulated in the tumors (FDR < 0.0069).

**Fig. 5:**
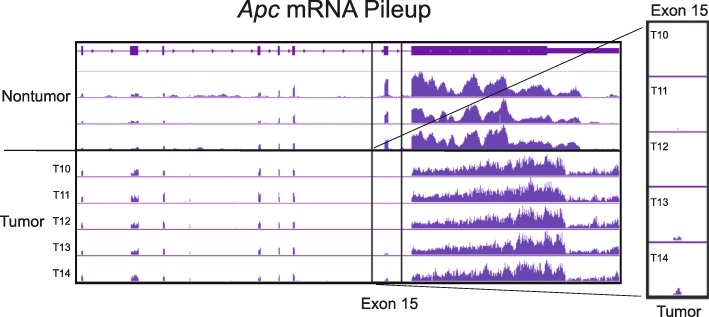
The adenomas selectively express mutated adenomatous polyposis coli (*Apc*) transcripts originating from a recombined transgene. The epigenetic loss of heterozygosity of *Apc* was apparent in the mRNA of the adenomas, which are missing exon 15 from Apc mRNA transcripts. RNA sequencing reads from adjacent nontumor tissue (top three rows) and adenoma (bottom five rows) are shown. The putative mechanism of Apc loss in these adenomas appears to proceed via epigenetic silencing of the wild-type allele and selective transcription of the mutant *Apc* gene, which lacks exon 15 after Cre recombinase- induced recombination under the control of the leucine-rich repeats and immunoglobulin-like domains 1 (*Lrig1*) promoter. The purity of the tumor samples is demonstrated by the complete lack of *Apc* exon 15 from tumors T10, T11, and T12. Tumors T13 and T14, which have retained some expression of *Apc* exon 15, were found to have gene expression patterns most similar to wild-type cells based on RNA-Seq

The tumors consistently displayed abnormal splicing patterns in several RNA-binding genes (Fig. 5). Compared to those in wild type mice, the tumor transcripts displayed a loss of intron retention in CRC-associated genes. Intron retention is a common feature of tumor genomes, but intron loss has been observed in breast cancer [47]. This observation suggests that modifications in post-transcriptional gene regulation are exploited by tumor cells in order to gain additional selective advantages against the host.

### Transcriptional silencing of wild type *Apc*

**Fig. 6:**
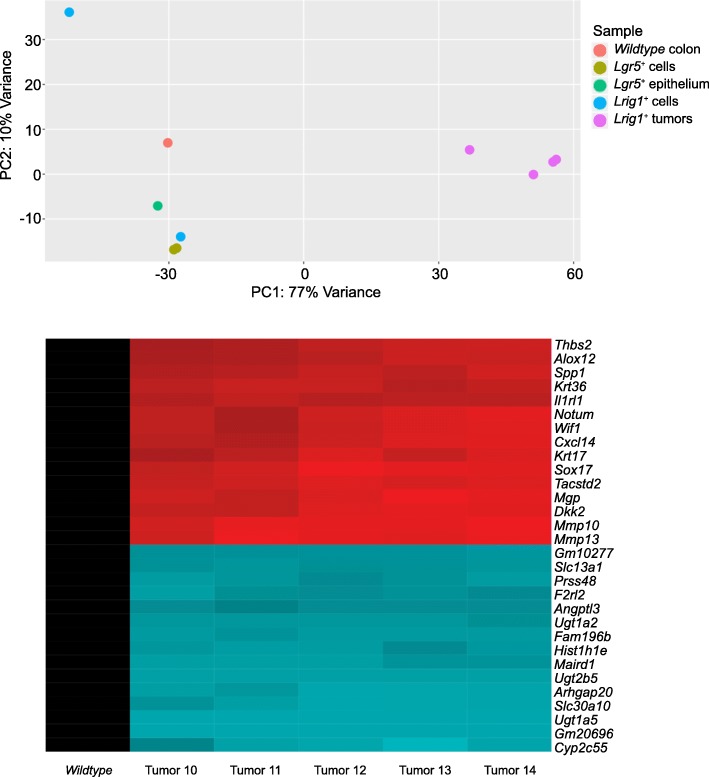
The tumors exhibited substantial transcriptome similarity to each other. Top: Principal components analysis of tumor transcriptome data compared to colons from untreated wild type mice. Tumor and wild type colon transcriptomes form distinct groups based on principal components analysis. Bottom: Heatmap visualization of highly significant differentially-expressed genes in the colonic adenomas. Rows are genes and columns are tumors. Genes colored red were up-regulated (top 15 rows) and genes colored blue were down-regulated (bottom 15 rows). Expression levels were normalized against that in the wild-type colon sample (black column on left). Many genes down-regulated are involved in detoxification of exogenous carcinogens

In contrast to previous reports, LOH of *Apc* was not observed in tumor exome DNA (Fig. 6). Allelic loss is typically considered to occur when the amount of tumor DNA is 50% or less of that obtained from heterozygous tissue due to the high risk of contamination from wild type cells. However, in this case, the tumor cells contained ~ 50% of the adjacent normal DNA, which was assumed to be homozygous for *Apc* since tamoxifen-induced Cre recombination in the colonic epithelium of these mice is a very rare event*.* No spontaneous somatic mutations were identified in the *Apc* gene that were of meaningful quality or significance. No genetic copy number variations or insertions or deletions of *Apc,* or any other gene, were readily apparent in the data.

**Fig. 7:**
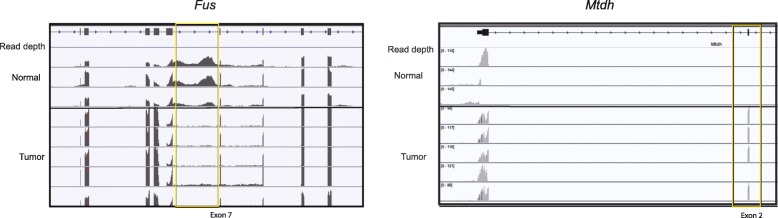
Colonic adenomas resulting from inducible loss of adenomatous polyposis coli (*Apc*) in leucine-rich repeats and immunoglobulin-like domains 1 (*Lrig1*)+/− stem cells have abnormal splicing patterns. The tumors consistently displayed abnormal patterns of intron retention in several RNA-binding genes. The Lrig1-(*Apc*-Flox) tumor transcriptome is characterized by a loss of intron retention in RNA-binding genes. Abnormal patterns of gene splicing were observed throughout the mRNA pileups of genes involved in RNA-binding, including the genes FUS RNA-binding protein (FUS) (left) and metadherin (Mtdh) (right). RNA sequencing reads from matched adjacent nontumor (‘normal’) tissue (top) and adenoma (bottom) are plotted

Unlike the exomic DNA, the tumor mRNA displayed a clear lack of functional *Apc* across the entire dataset (*n* = 5/5 tumors). The adenomas expressed exclusively *Apc* transcripts lacking codon 580 in exon 15 (Fig. 7), which is the same sequence as the original *Apc* transgene after tamoxifen-induced recombination has occurred. These results imply that the tumors have acquired the ability to selectively express nonfunctional *Apc* transcripts. This epigenetic phenomenon appears to have occurred across all tumors, implying that the LOH of *Apc* was an early event of adenoma formation.

## Discussion

Evidence of early genomic changes were apparent throughout the exomes and transcriptomes of the tumors sequenced in this study. The *Lrig1-CreERT2/+;Apcfl/+* tumors displayed a hypermutated phenotype; each tumor exome contained a unique profile of ~ 100 mutated alleles predicted as ‘high impact’ based on amino acid sequences. The tumors contained a very high incidence of C:G > A:T transversion point mutations, which result from the oxidation of guanine to form guanine adducts, and typically occur in smoking-induced lung cancer and stomach cancer caused by *H. pylori*.

The hypermutated phenotype of *Lrig1-CreERT2/+;Apcfl/+* tumors could have resulted from an increased rate of mutation or a reduced rate of DNA repair. The glucuronidation pathway was strongly downregulated in the tumor transcriptomes, and many inflammation markers were upregulated. It is unclear if these gene expression changes were the cause or result of the high incidence of mutations in the tumor. Microbial-produced reactive oxygen and nitrogen species (ROS and RNS) are a likely source of guanine adducts and C:G > A:T transversion point mutations in the colon. The high incidence of *Lrig1-CreERT2/+;Apcfl/+* adenomas localized to the distal colon might be explained by the high levels of ROS inherent to the distal colon and rectum.

Genetic heterogeneity was observed in tumors from the same mouse (intratumoral heterogeneity), as well as from sibling mice (intertumoral heterogeneity). Interestingly, in contrast to their variable genomes, the tumor transcriptomes and splicing patterns were strikingly uniform across all samples. The uniformity of the tumor transcriptomes suggests that the adaptation of an invasive phenotype resulted from convergent evolution. The lack of uniformity in the tumor genomes compared to the transcriptomes implies that expressed RNA and proteins may represent better drug targets than mutations, due to the fact that tumor transcriptomes are more uniform and predictable than tumor genomes.

The *Lrig1-CreERT2/+;Apcfl/+* mouse model of CRC is notable for its high penetrance and rapid onset after the loss of just a single copy of *Apc.* The extremely high tumor burden observed in *Lrig1-CreERT2/+;Apcfl/+* mice is especially striking considering that prior to the spontaneous loss of an *Apc* allele, tamoxifen-induced Cre-recombination of the other Apc allele, must first occur in the same cell under the control of the *Lrig1* promoter, which is also a rare event. Spontaneous LOH of *Apc* in humans and mice tends to occur much more slowly, generally in the order of several months to years. For example, human familial adenomatous polyposis intestinal tumors typically become malignant at around age 40 years.

In contrast to previous reports, a genetic loss of heterozygosity (LOH) of Apc was not observed in the *Lrig1-CreERT2/+;Apcfl/+*tumors. This fact is unsurprising when one considers the extremely low theoretical likelihood of multiple spontaneous somatic LOH events at a single genetic locus multiple times in one colon over the span of a few months. It is worth noting that in the original report on these tumors, a standard polymerase chain reaction genotyping assay was used to detect the LOH of *Apc*, which is an archaic and much less sensitive technique for genotyping compared to DNA sequencing.

Despite the normal appearance of the remaining *Apc* alleles in the tumor exome DNA, there was clear evidence of an epigenetic (‘above the genes*’*) LOH of exon 15 of *Apc* observed in the tumor mRNA. Unlike the tumor DNA, the *Apc* mRNA transcripts expressed by the tumor cells clearly displayed a copy number loss of exon 15. The expressed *Apc* transcripts had a genetic sequence identical to that of the original *Apc* transgene following Cre-induced recombination, indicating that only the mutant transgene is being expressed by the tumor cells. The assumed mechanism responsible for the lack of wild type *Apc* expression is transcriptional silencing of the single wild type copy of *Apc* remaining after engineered recombination of the *Apc* transgene. It is possible that the double heterozygosity of *Lrig1* and *Apc* act synergistically with *Lrig1* haploinsufficiency to increase the possibility of transcriptional silencing of wild type *Apc*.

The proposed mechanism of colonic adenoma formation in the *Lrig1-CreERT2/+;Apcfl/+* model is the following: Transcriptional silencing of *Apc* leads to a decrease in goblet cells and a corresponding increase in inflammatory reactive oxygen species (ROS) in the distal colon. Somatic C > A mutations resulting from increased inflammation provide the raw genetic material required for adenoma formation and progression. Natural selection of growth-promoting mutations leads to convergent evolution towards a transcriptome marked by dysregulated splicing and altered gene expression resulting in an invasive phenotype.

The results of this study demonstrate that the genomic events leading to tumor growth and invasion can happen early in tumorigenesis and that tumors can generate large pools of mutations early in the course of disease progression. The selection of advantageous mutations is a dynamic process, changing throughout the course of tumorigenesis. The ability of a tumor to create and store mutations in a reservoir for later use provides an advantage to the tumor by optimizing its ability to dynamically modulate the selection of oncogenic mutations in a context-dependent manner throughout disease progression. Mutational reservoir creation provides tumors with the ability to conditionally express somatic mutations which could ultimately leading to tumor metastasis and drug resistance.

In order to develop therapeutic treatments that are less susceptible to drug resistance, it is important to understand the mechanisms underlying tumor formation and evolution. Going forward, mutational processing mechanisms altered during early tumorigeneses make attractive therapeutic targets since they should theoretically be present in a large proportion of tumor cells and they are the drivers of tumor growth. Additional studies are needed in order to understand the mechanisms underlying the processes of mutation generation in tumors.

## Conclusions

Understanding the process of mutation generation during tumorigenesis can provide insights into the mechanisms underlying cancer cells’ ability to evolve and grow. *Lrig1-CreERT2/+;Apcfl/+* tumors exhibited transcriptional silencing of *Apc* and contained a high incidence of C > A mutations cause by increased inflammation. In contrast to their variable genomes, the tumor transcriptomes and splicing patterns were strikingly uniform across all tumors, and likely represent more predictable cancer biomarkers.

## Supplementary information

**Additional file 1.** Somatic mutations identified in *Lrig1-Cre/+;Apcfl/+* colonic adenomas.

**Additional file 2.** Annotated RNA-Seq results for in *Lrig1-Cre/+;Apcfl/+* colonic adenomas vs. wildtype colon.

**Additional file 3.** Raw RNA-Seq results for in *Lrig1-Cre/+;Apcfl/+* colonic adenomas vs. wildtype colon.

## Data Availability

The datasets used and/or analyzed during the current study are available from the corresponding author on reasonable request.

## Abbreviations

CRC: Colorectal cancer
LOH: Loss of heterozygosity
ROS: Reactive oxygen species
